# Variable Gut Function Recovery After Right vs. Left Colectomy May Be Due to Rectosigmoid Hyperactivity

**DOI:** 10.3389/fphys.2021.635167

**Published:** 2021-02-23

**Authors:** Sean Ho Beom Seo, Ian Bissett, Gregory O’Grady

**Affiliations:** Department of Surgery, University of Auckland, Auckland, New Zealand

**Keywords:** ileus, colectomy, recovery, electrophysiology, prolonged ileus, cyclic motor patterns

## Abstract

It is established that gut function recovers slower after right vs. left colectomies with higher rates of prolonged post-operative ileus (PPOI), but the reason is unclear. Development of PPOI is multifactorial. A recent manometry study in right colectomy patients showed that the distal colon becomes hyperactive after surgery with predominantly cyclic motor patterns (CMPs). In this perspective, we evaluate the hypothesis that the slower gut recovery after right hemicolectomy could be induced by a functional obstruction due to hyperactive CMPs.

## Introduction

**FIGURE 1:**
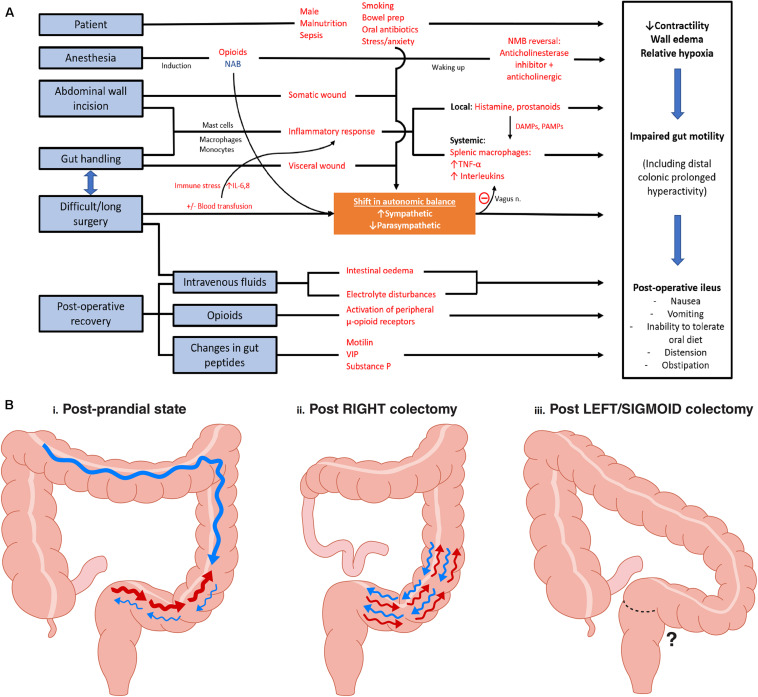
**(A)** Peri-operative factors and pathophysiology in development of postoperative ileus. Red words indicate factors that increases likelihood of ileus. NAB, neuroaxial blockade; NMB, neuromuscular blockade; DAMPs, damage-associated molecular patterns; PAMPs, pathogen-associated molecular patterns; VIP, vasoactive intestinal peptide. Adapted and modified from Vather et al. (2014). **(B)** Rectosigmoid regional specific activity: Observations made from high resolution manometry studies. **(i)** Post-prandial state: Following a 700 kcal meal, significant increased number of retrograde contractions were observed at the rectosigmoid region (CMPs in a 2 h period: Pre-prandial 3.9 ± 3.8 vs. post-prandial 84.9 ± 26.0, *P* < 0.05). Active state comprised of 27% of study duration. HAPS* were noted proximally. **(ii)** Following right colectomy, an intense and sustained (94% of study duration) hyperactive CMP** was noted in all eight patients studied. **(iii)** Post Left/Sigmoid Colectomy: the sigmoid and rectosigmoid regions are resected, meaning that the hyperactive CMP cannot occur; however, this state has not yet been studied using reliable motility mapping techniques. *HAPS, High amplitude propagation sequences; **CMP, Cyclic motor patterns defined as repetitive pressure/contractile events occurring at 2–6 cycles/min regulated by external innervation. Adapted from findings in publications Lin et al. (2017b) and Vather et al. (2018a).

Surgery impairs colonic function both acutely and chronically. One in five patients undergoing colorectal surgery suffer delayed colonic functional recovery for more than 3 days, which is termed prolonged post-operative ileus (PPOI). PPOI, which is understood to be multifactorial (Figure 1A), increases costs of care by 70% through longer inpatient stay, increased investigations, and from managing the complications of ileus such as increased thromboembolic events and prescription of parenteral nutrition (PN) (Mao et al., 2019).

Conventional multimodal enhanced recovery after surgery (ERAS) protocols have helped to reduce length of stay (LOS), morbidity, and rates of PPOI. However, isolating the specific effects of individual components of the protocols on gut recovery is difficult. Adaptations of ERAS have also varied not only between countries but also individual hospitals, which make comparative analyses challenging. Most significantly, the actual mechanisms of post-operative gut dysfunction remain incompletely understood. An improved understanding of colonic post-surgical pathophysiology would enable better identification of “at risk” patients and enhanced preventative and therapeutic strategies.

In recent years, the use of high-resolution colonic manometry has enabled improved characterization of colorectal motility patterns. One of the important discoveries concerns rectosigmoid function. It has been suggested that the most distal region of the colon has a specialized “rectosigmoid brake” role, whereby retrograde CMPs feature prominently after meals, at a rate of approximately 2–4 cycles per minute, to limit rectal filling and thereby potentially contributing to continence (Rao and Welcher, 1996; Lin et al., 2017b).

It was recently found that rectosigmoid cyclic motor activity becomes dramatically more active peri-operatively and that this hyperactivity is sustained for at least 16 h following right hemicolectomy. Another interesting finding was that the CMPs in the distal colon increased in intensity as the time to entering surgery approaches in a fasted and pain-free state. Vather et al. suggested pre-operative anxiety may be responsible, via sympathetic nervous output and its subsequent effect on gut motility (Vather et al., 2018a).

Three principles are becoming evident:

(i) The surgical recovery of right colectomies is delayed in comparison to resections of the contralateral side (Yuan et al., 2018; Garfinkle et al., 2019).
(ii) A rectosigmoid brake, possibly mediated by CMPs, likely acts as a gatekeeper to the passage of feces into the rectum (Lin et al., 2017b).
(iii) Post-operatively, rectosigmoid CMPs become hyperactive for at least 16 h (Vather et al., 2018b).

This perspective evaluates the hypothesis that the time to resolution of rectosigmoid hyperactivity impacts gut recovery times after colectomy. It is proposed that resections of the left colon (including anterior resection) may result in relatively less post-operative CMP hyperactivity, because the responsible anatomical regions are resected. Therefore, in this framework, the specific motor patterns of the rectosigmoid colon could be responsible for the discrepancy in recovery rates between right and left sided resections. This concept is summarized in Figure 1B. The background, rationale, and potentially implications of this proposal are evaluated.

## Gut Recovery Is Different After Right vs. Left Sided Colonic Resection

A preferred biomarker for comparisons of physiological gut recovery after surgery is time to oral diet and first bowel motion (i.e., “GI-2” composite criteria), because it correlates with transit (Van Bree et al., 2010; Vather et al., 2018a). In addition, a distinction between POI and PPOI was offered in a consensus article in 2013, though the terms remain debated (Vather et al., 2013). Under this framework, POI indicates an “obligatory” delay to complete GI tract recovery within a consensus normal timeframe of <4 days. Delay of gut function for 4 days or more defines PPOI, an abnormally extended course of gut dysfunction. “Primary” and “secondary” PPOI have also been defined, with secondary PPOI being a sequalae of complications, primarily sepsis. Left sided resections entail a higher rate of severe post-operative sepsis (requiring interventions including either drainage or return to theater), affecting recovery and the incidence of POI (Moghadamyeghaneh et al., 2016). It is necessary to account for these issues to achieve representative data on physiological recovery patterns after right vs. left sided colectomies.

With these considerations accounted for, a considerable number of studies in the literature now have robustly shown that right sided colectomies recover more slowly than left sided resections in modern colorectal practice. In 2016, a Swiss study revealed a threefold increase in the rate of ileus with right sided resections (24 vs. 8%, *P* = 0.002) along with longer LOS (6 vs. 5 days, *P* = 0.02), with compliance to ERAS protocols being equivalent (Kummer et al., 2016). A 2017 study presented a nomogram for risk of ileus in patients undergoing colectomy, employing data from the American College of Surgeons, National Surgical Quality Improvement Program (NSQIP) database. Right colectomy with ileo-colic anastomosis (described as “partial colectomy with removal of terminal ileum and ileocolostomy”) was formally acknowledged as a risk factor for ileus, entailing a risk ratio (RR) of 1.218 (*P* = 0.003), when compared with “partial colectomy with anastomosis” for development of PPOI. By comparison, “partial colectomy with low pelvic anastomosis” did not lead to an increase in ileus (RR 0.992, *P* = 0.91) (Rencuzogullari et al., 2017). These notable series highlight the mounting evidence that right colectomies have a slower rate of recovery post-operatively.

In 2019, a further study from the United States using the NSQIP database compared nearly 13,000 patients undergoing oncological elective resections, and a higher incidence of primary PPOI in the right colectomy group (11.5 vs. 8.8%, *P* < 0.001) was detected (Garfinkle et al., 2019). In this study, patient data was coarsened-exact-matched for confounders such as age, sex, ASA score, operative approach, and transfusion rates.

Several potential mechanisms were offered in these studies for the slower recovery of right-sided resections, including that differential activation of retroperitoneal nerves, method of anastomosis (handsewn ileocolic vs. stapled colo-colic/colorectal), and the choice of iso- or antiperistaltic ileocolic anastomosis may impact on gut functional recovery as well (Campana et al., 2017; Ibanez et al., 2019). As per Figure 1B, we propose an alternative mechanism, which although currently quite speculative has emerging physiological evidence, which is that the intact section of the distal colon may be physiologically responsible due to hyperactive CMPs.

## Mechanisms of Ileus

Prevention of ileus has been an area of considerable attention, driven by a desire for improved patient outcomes as well as greater efficiencies by reducing LOS in hospitals and costs of care. The expected length of stay following bowel surgery has been progressively reduced from a traditional window of 10+ to 3–5 days or less. The synergy of minimally invasive surgery and ERAS have achieved this efficiency in the post-operative care setting, and progressively moving toward even shorter LOS. However, PPOI still occurs at a rate of 10–25% in the current era in many published settings. Understanding the pathophysiology that underpins the development of PPOI will help identify the areas where interventions can be effectively targeted. Hyperactive CMP activity in post-operative patients is a recent physiological finding from which our perspective has developed. This perspective aims to describe how post-surgical dysmotility (and potentially an underlying autonomic imbalance) may be implicated in the occurrence of PPOI.

Gut recovery after colonic resection is understood to occur in phases. It is generally understood that small bowel typically recovers within 24 h, stomach 24–48 h, and the colon the slowest at more than 48 h. If GI-2 is not reached before day 4, then a consensus paper has proposed that PPOI can be diagnosed by fulfillment of two of the following five criteria (Vather et al., 2013):

(1) Nausea or vomiting (over preceding 12 h).
(2) Inability to tolerate a solid/semi-solid diet over the 2 preceding mealtimes.
(3) Absence of flatus and stool over last 24 h.
(4) Abdominal distension.
(5) Radiologic confirmation of ileus on plain x-ray or CT in last 24 h.

Once PPOI is clinically recognized, treatment is mainly supportive, including by nasogastric tube placement, supporting intravenous fluids, and PN if required. Secondary causes of ileus should be excluded, such as intra-abdominal sepsis and anastomotic leak and electrolytes normalized. PPOI is the commonest cause of acute intestinal failure requiring PN, adding to the substantial healthcare costs of ileus (Brokenshire et al., 2009).

Factors in ileus development are varied and complex (see Figure 1A). Appreciation of the autonomic nervous system’s (ANS) role in inflammatory, immunological, and neuro-modulative influences of gut function has expanded. Principle inciting factors in ileus are breaches of the parietal and visceral peritoneum (mandated in colonic surgery), and documented risk factors include open surgery, length of wound greater than 10 cm, extensive bowel handling, difficult surgery, red cell transfusion requirement, and the patient factor of being male (Vather et al., 2015).

Traditionally, two distinct phases of ileus pathogenesis have been proposed: a quick-onset neurogenic phase of overexcited inhibitory splanchnic reflexes at the spinal level, preceding a delayed but longer inflammatory phase. More recently a cholinergic anti-inflammatory pathway has been discovered implying a second and larger influence of the ANS (Vather and Bissett, 2013; Chapman et al., 2018).

The inflammatory phase initiates with somatic and visceral wounds. Local factors (i.e., activation of mast cells within the peritoneum and resident muscularis externa macrophages) and a secondary immune-neurological interplay between nociceptive afferents and pro-inflammatory cytokine production (e.g., TNFα and interleukins) are responsible for inducing a global myoenteric dysfunction (Wang et al., 2003; Vather and Bissett, 2013). It is well accepted that imbalance of sympathetic and parasympathetic output is responsible for colonic dysmotility, but also, they ANS exerts influence high in the inflammatory cascade, which has downstream effects on gut function. Intra-operative electrical stimulation of the vagus nerve trunks has shown potential to both decrease inflammatory cytokine production and reduce the occurrence of PPOI, which may work synergistically (Stakenborg et al., 2017).

An example of the autonomic nervous system’s dual effects on colonic function (direct innervation and influence via inflammatory pathways) may be found with neuroaxial blockade (NAB). Post-operative epidural infusions/intermittent boluses were historically used in ERAS protocols in colorectal surgery. Operative factors such as peritoneal irritation, visceral disruption, as well as peri-operative anxiety and pain activate inhibitory sympathetic reflexes that affect bowel motility by preventing the release of acetylcholine, the main neurotransmitter for peristalsis. NAB affects the somatic innervation below a certain level of the spinal cord, as well as the sympathetic tone corresponding to the level of blockade. There have been no studies correlating peri-operative sympathetic activity and bowel recovery; however, there are numerous studies comparing NAB, bowel function, and length of stay. A Cochrane review in 2016 reported evidence for faster gastrointestinal (GI) recovery with the use of epidural analgesia for patients undergoing open surgery (Guay et al., 2016). A more recent meta-analysis identified eight randomized controlled studies (three laparoscopic), five of which demonstrated faster gut functional recovery with epidural anesthesia in comparison with opioid patient controlled analgesia (Chapman et al., 2018). Epidurals are now used less commonly due to the rise of minimally invasive surgery.

In summary, imbalance of the ANS is likely a key influence in the development ileus, and this role is extended in an emerging proposal for the mechanism of delayed recovery after right hemicolectomy, as detailed in the next section.

## Colonic Physiological Investigations

Early low-resolution manometry studies (i.e., incorporating a low number of manometry sensors) identified high amplitude propagating sequences (HAPS). These pressure waves traveled antegrade for long distances and are recognized to be responsible for colonic mass movements. The advent of high-resolution manometry (HRM) in the past decade has robustly identified additional motility patterns that are shorter and in lower amplitude occurring both antegrade and retrograde forms that occur multiple times a minute, particularly CMPs. CMPs are thought to be related to interstitial cells of Cajal (ICC) pacemaker activity but requiring co-regulation from the enteric and autonomic nervous systems as well as integration by smooth muscle. In *ex vivo* preparations, artificial electrical field stimulation can regulate CMP activity not only in amplitude and frequency, but also by direction of propagation (Rosli et al., 2020). Currently there are no established large animal models to study CMPs in the context of surgery. A systematic review of publications that studied the colonic electromechanical abnormalities underling postoperative ileus identified 19 studies, most of which were low resolution, including two animal studies performed in the 1970s and 80s using low resolution bipolar devices that failed to detect and describe colonic CMPs (Wells et al., 2019a).

**FIGURE 2:**
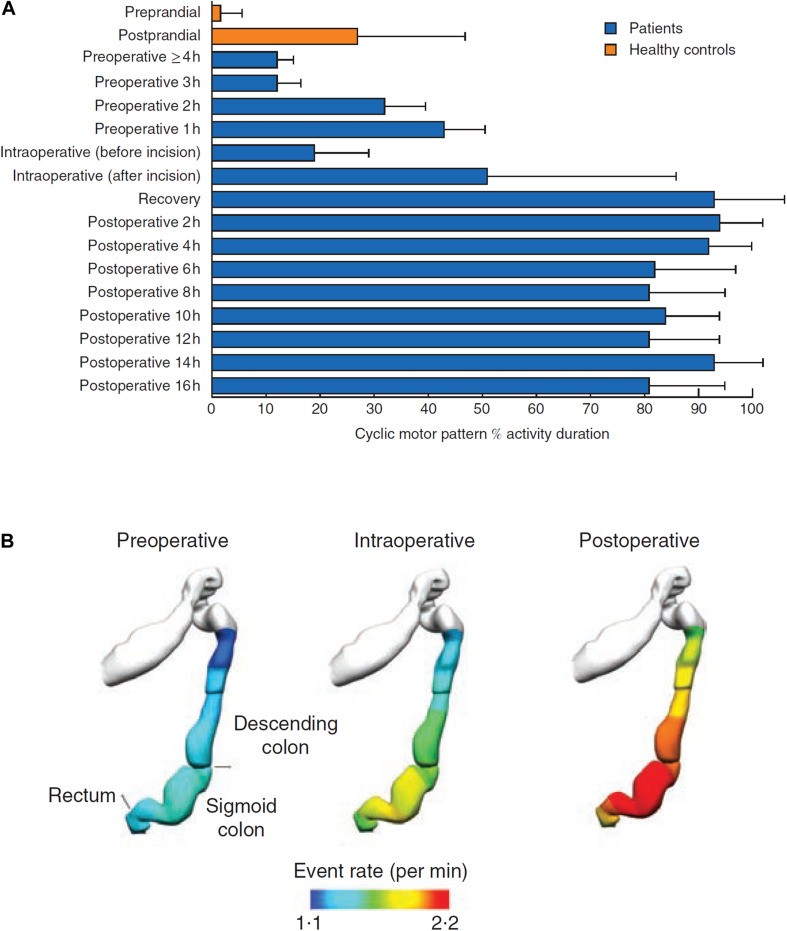
**(A)** In all patients, there was a marked increase in the occurrence of cyclic motor patterns that began before operation, increased during surgery, and was largely maintained after operation (*P* < 0.001, repeated-measures ANOVA). **(B)** Anatomical registration of the event rate distribution in a colonic geometry model, based on the estimated catheter insertion position. The colors represent the mean number of propagating events per minute over the entire recording period. The cyclic motor pattern was most active in the sigmoid colon, reproduced with permission, from “Hyperactive cyclic motor activity in the distal colon after colonic surgery as defined by high-resolution colonic manometry,” Vather et al. (2018a).

A recent HRM study found that conventional notions that colonic motor activity becomes quiescent following colonic surgery were incorrect. In 8 right colectomy/ileocolic resection patients, the data revealed a hyperactivity of CMPs in the rectosigmoid region, lasting for > 16 h post-operatively (Figures 2A,B; Lin et al., 2017a; Vather et al., 2018a; Wells et al., 2019b). The pathophysiology is unclear but was proposed to relate to an over-expression of an innate motility pattern normally observed post-prandially and co-regulated by the ANS. Vather et al. further suggested that excessive sympathetic nerve activity post-operatively could inhibit enteric nerves that are themselves inhibitory, thereby allowing the unsuppressed expression of the hyperactive CMPs; although this putative mechanism current remains speculative and other neural or hormonal mechanisms are alternative contributors (Vather et al., 2018a).

This pronounced hyperactivity has only been measured out to 16 h in studies to date, and direct correlation with motility or with post-operative clinical gut recovery (e.g., GI-2) has not yet been performed. To induce clinically significant delayed bowel recovery, the activity would have to continue for up to several days after right hemicolectomy, correlating with motility and clinical recovery (duration of obligatory ileus/POI), and its duration would be variable between patients. These requirements now await further evaluation.

In indirect support of the hypothesis, Yuan et al. in a prospective study showed not only that right sided resection patients were significantly slower to recover GI function (median 2.5 vs. 4 days, *P* = 0.03) but also that the longer the length of bowel resected on the left side (i.e., removing more colon where hyperactivity occurs) positively correlated with faster recovery, whereas the right-sided resections were slower to recover when the resections were more extensive (Yuan et al., 2018). This data reinforces the possibility that resection of the anatomical regions primarily responsible for CMPs may ameliorate the effect of hyperactivity on delayed gut recovery (Figure 1B).

## Testing the Hypothesis

To test this hypothesis further, a direct observation of colonic hyperactivity would be required throughout the period of colonic recovery post-operatively. Traditionally this was measured by HRM. However, in our experience HRM is not well tolerated by patients for longer than 24 h, especially when their mobilization is normally encouraged in ERAS protocols. There is also an inherent risk in instrumentation left-sided resections with an anastomosis, limiting post-operative use of HRM in evaluating left hemicolectomies. Nevertheless, longer-duration HRM studies following right colectomies are currently ongoing.

These issues could potentially be mitigated in the future using an emerging novel motility mapping technology called electrocolonography (EColG). A proposed multichannel body surface mapping method has been presented that could achieve this, involving the use of a high-resolution electrode array, providing a non-invasive alternative to HRM. As EColG is a novel technique, there are limited published data to date; however, it has been specifically validated to detect cyclic motor activity and methods are progressing. Early evidence shows that the same 2–4 cycle per minute CMPs can be detected using EColG, including the increased post-prandial activity (Erickson et al., 2020). If validated in post-operative patients, EColG could be applied to achieve clinicopathological correlation of the hyperactive CMP, including detailed comparisons of right vs. left colectomy. Within our unit, work is currently underway to record simultaneous HRM and body surface recordings with meal-tests on non-operative participants to further validate the non-invasive detection of colonic activity.

In right colectomy patients, EColG could be expected to demonstrate varying durations of hyperactivity occurring in the rectosigmoid region. For left sided resections, depending on how much of the rectosigmoid junction has been resected, reduced activity would be anticipated, as recently revealed in patients suffering chronic Low Anterior Resection Syndrome (Keane et al., 2020).

## Perspectives on the Proposed Mechanism

If confirmed, a positive correlation between PPOI and rectosigmoid hyperactivity would challenge one current paradigm in ileus pathophysiology, while presenting a non-invasive biomarker of colonic recovery to inform future studies in therapeutics modulating CMP hyperactivity. Better blockade of somatic and autonomic nociceptive afferent pathways to reduce the sympathetic output could also be beneficial.

Despite the growing body of knowledge on the differences between right and left sided colectomy patients in terms of demographics, tumor variables, complication profiles, and recovery patterns, preventative and treatment approaches to PPOI have lagged. Furthermore, various treatments for PPOI have been tested without clear reference to mechanisms of action. Consequently, most ERAS protocols still currently have a non-specific approach, without recognition of the location and extent of the resection and individual patient factors. An improved understanding of the specific motility consequences of different anatomical regions of the colon could personalize perioperative expectations and treatment in future.

A non-invasive method to identify biomarkers of colonic recovery could also help researchers to delineate positive and negative factors involved in prolongation of ileus, detect early signs for PPOI, and tailor ERAS protocols to be more side-specific. In the future, if clinically implemented, the real time interpretation of colonic motor function could also, with a degree of statistical certainty, allow patients to be discharged sooner, i.e., if hyperactivity had settled but bowels had not yet opened.

## Conclusion

In conclusion, early emerging evidence suggests that post-operative rectosigmoid CMP hyperactivity could hinder the return of gut functional recovery and explain mechanisms of delayed recovery after right vs. left colectomy. More data is certainly required. However, if proven, this paradigm could motivate a reconsideration of optimal approaches to the peri-operative care of post-colectomy patients. Specifically, a novel biomarker of colonic recovery could help to achieve a shift toward personalized care based on actionable and objective measurements of colonic function. Ultimately, further research will determine whether hyperactivity is a clinically useful biomarker of PPOI.

## Data Availability Statement

The original contributions presented in the study are included in the article/supplementary material, further inquiries can be directed to the corresponding author/s.

## Author Contributions

SS, GO’G, and IB developed the presented idea. SS performed the literature review, designed and adapted the figures, and wrote the manuscript with support from GO’G and IB. All authors contributed to the article and approved the submitted version.

## Conflict of Interest

GO’G is a Director of the University of Auckland spin-out company Alimetry Ltd. The remaining authors declare that the research was conducted in the absence of any commercial or financial relationships that could be construed as a potential conflict of interest.
